# Comparison of Two High-Throughput Assays for Quantification of Adenovirus Type 5 Neutralizing Antibodies in a Population of Donors in China

**DOI:** 10.1371/journal.pone.0037532

**Published:** 2012-05-24

**Authors:** Qiang Liu, Jianhui Nie, Weijin Huang, Shufang Meng, Baozhu Yuan, Dongying Gao, Xuemei Xu, Youchun Wang

**Affiliations:** 1 Key Laboratory of the Ministry of Health for Research on Quality and Standardization of Biotech Products, Department of Cell Biology, National Institutes for Food and Drug Control, Beijing, China; 2 Department of Biophysics and Structural Biology, School of Basic Medicine, Peking Union Medical College, Institute of Basic Medical Sciences, Chinese Academy of Medical Sciences, Beijing, China; 3 Beijing Blood Center, Beijing, China; Naval Research Laboratory, United States of America

## Abstract

**Background:**

The presence of various levels of Adenovirus serotype 5 neutralizing antibodies (Ad5NAb) is thought to contribute to the inconsistent clinical results obtained from vaccination and gene therapy studies. Currently, two platforms based on high-throughput technology are available for Ad5NAb quantification, chemiluminescence- and fluorescence-based assays. The aim of this study was to compare the results of two assays in the seroepidemiology of Ad5NAb in a local population of donors.

**Methodology/Principal Findings:**

The fluorescence-based neutralizing antibody detection test (FRNT) using recombinant Ad5-EGFP virus and the chemiluminescence-based neutralizing antibody test (CLNT) using Ad5-Fluc were developed and standardized for detecting the presence of Ad5NAb in serum samples from the population of donors in Beijing and Anhui provinces, China. First, the overall percentage of people positive for Ad5NAb performed by CLNT was higher than that obtained by FRNT (85.4 vs 69.9%, p<0.001). There was an 84.5% concordance between the two assays for the 206 samples tested (144 positive in both assays and 30 negative in both assays). All 32 discordant sera were CLNT-positive/FRNT-negative and were confirmed positive by western blot. Secondly, for all 144 sera positive by both assays, the two assays showed high correlation (r = 0.94, p<0.001) and close agreement (mean difference: 0.395 log_10_, 95% CI: −0.054 log_10_ to 0.845 log_10_). Finally, it was found by both assays that there was no significant difference observed for titer or prevalence by gender (p = 0.503 vs 0.818, for two assays); however, age range (p = 0.049 vs 0.010) and geographic origin (p = 0.007 vs 0.011) were correlated with Ad5NAb prevalence in northern regions of China.

**Conclusion:**

The CLNT assay was relatively more simple and had higher sensitivity than the FRNT assay for determining Ad5NAb titers. It is strongly suggested that the CLNT assay be used for future epidemiological studies of Ad5NAb in other localities.

## Introduction

Adenoviruses include a large family of non-enveloped, double-stranded DNA viruses, which generally cause respiratory diseases and ocular diseases in humans of all age groups in addition to gastrointestinal disorders in children [1]. Adenovirus serotype 5 (Ad5) is widely used as a vehicle for vaccine delivery for the treatment of infectious disease and cancer [2]–[4]. However, the efficacy of Ad5 vectors has been limited in humans because exposure to natural Ad5 infections results in a high percentage of potential vaccinees having neutralizing antibodies against Ad5 (Ad5NAb), particularly in the developing world [5]–[9]. Therefore, it is necessary to determine the prevalence of Ad5NAb in a study population before the administration of Ad5 vector-based products [10], [11].

Ad5NAb titer is typically obtained by transgene expression inhibition and replication inhibition with plaque scoring [12]. Quantitative analysis based on the transgene expression inhibition is supported by previous data that showed the number of recombinant virus particles bound to cells was directly proportional to transgene expression [13]. A series of enzyme-activated chemiluminescence-based neutralizing antibody detection test (CLNT) have been developed for the detection of Ad5NAb, including firefly luciferase (Fluc), β-galactosidase, and secreted alkaline phosphatase reporter genes [14]–[17]. Enhanced green fluorescent protein (EGFP) has also been widely used as a reporter gene in transgene expression inhibition assays, known as the fluorescence-based NAb detection test (FRNT) [18], [19]. Currently, most available Ad5NAb assays have employed CLNT and FRNT techniques that provide many benefits, including improved dynamic range, simplicity, and significant increase in laboratory throughput.

Several studies have been conducted with CLNT and FRNT for their efficacies of detecting Ad5NAb. Rajendra et al [19] found 100% of sera from 114 representatives of an Indian adult population had different titers of Ad5NAb using FRNT assay, starting dilution at 1∶10. However, Caijun et al [15] investigated the epidemiology of Ad5NAb in healthy people in Guangzhou, southern China using a CLNT assay and found a lower seroprevalence (77.34%), starting dilution at 1∶18. These studies highlight that it is unknown how these two assays compare for measuring Ad5NAb levels in human sera. Therefore, differences in data obtained by these assays prove difficult to interpret and compare since the sensitivity of the assays or prior exposure to Ad5 infection likely influences each assay differently. In the present study, we describe a head-to-head comparison of the CLNT and FRNT assays using sera from healthy individuals in Beijing and Anhui provinces in northern China.

## Results

### Construction of Ad5-Fluc and Ad5-EGFP

**Figure 1:**
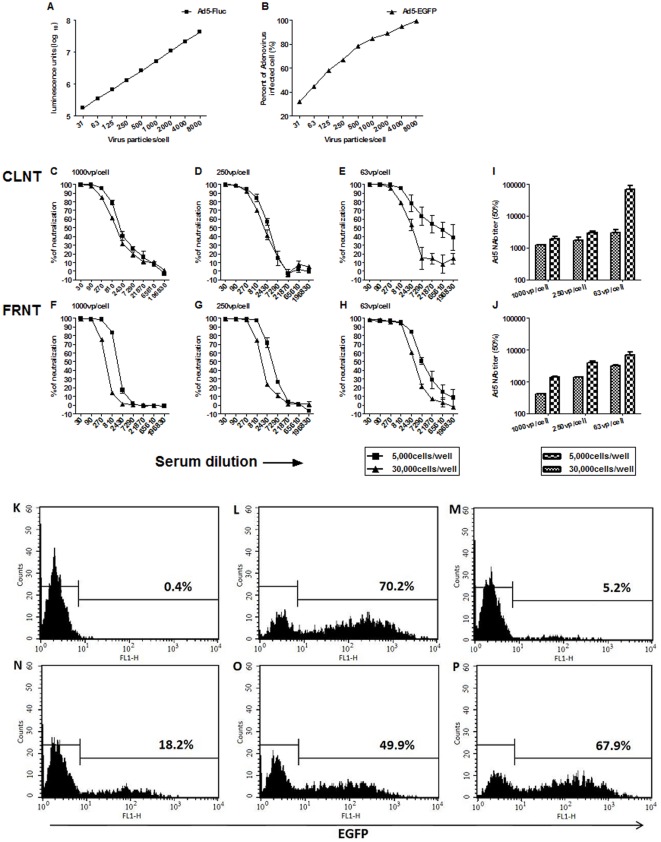
Optimization of conditions for CLNT and FRNT assays. (A) Dose-response between Ad5-Fluc virus inoculum and luciferase transgene expression. (B) Dose-response between Ad5-EGFP virus inoculum and percent of infected cells. The x-axis indicated virus concentration, ranging from 31–8000 VP/cell, added to 30,000 cells/well in a 96-well plate. Each data point represented the average of three experiments. (C–H) Different amounts of virus and cell density were used in the neutralization assay (▪, 5,000 cells/well; ▴, 30,000 cells/well), and the percentages of neutralization were shown for nine serial 3-fold dilutions of Ad5-vaccinated mouse serum, with CLNT (C–E) and FRNT (F–H) assays. Each data point represents the average of three experiments. (I–J) Ad5NAb titers determination with CLNT (I) and FRNT (J) assays. Each column represents the average of three experiments. (K–P) FACS analysis of Vero cells infected with Ad5-EGFP expressing the EGFP reporter gene. Incubation with optimized viral dose (250VP/cell) and cell density (30,000 cells/well) with a 810-fold (M), 2430-fold (N), 7290-fold (O), 21870-fold (P) dilutions of Ad5-vaccinated mouse serum or without serum (L) resulted in 5.2%, 18.2%, 49.9%, 67.9% or 70.2% EGFP-positive cells, respectively, and uninfected cell control (K) resulted in 0.4% EGFP-positive cells. The mean percentage of infected cells was shown in each plot. Representative data were shown for three independent experiments.

After 4–5 rounds of propagation in HEK293 cells, insertion of the Fluc and EGFP genes into the Ad5 genome, creating Ad5-Fluc and Ad5-EGFP, respectively, and were confirmed by polymerase chain reaction (PCR) and nucleotide sequencing. The amplified Fluc and EGFP products have 100% nucleotide identity to published sequences (GenBank Accession: EU921841 for Fluc, DQ768212 for EGFP). As shown in Figure 1A and 1B, the expression of luciferase and EGFP reporter genes were detected using the CLNT and FLNT assays. The Ad5-Fluc (lot number: 20101225) and Ad5-EGFP virus stocks (lot number: 20101227) were purified by cesium chloride gradient centrifugation, diluted in phosphate-buffered saline (PBS) to a final concentration of 1×10^12^ virus particles (VP)/mL, titrated using ultraviolet spectrophotometry, and stored at −80°C.

### Optimization of conditions for the neutralization assay

Vero cells were infected with different doses of Ad5-Fluc or Ad5-EGFP virus to determine the optimal concentration to be used in the neutralization assay. Figure 1A shows the direct correlation between Ad5-Fluc virus concentration of 31–8000 VP/cell and reporter transgene expression (r^2^ = 0.9998). However, Ad5-EGFP virus at 31–1000 VP/cell was shown to be better correlated with the percent of infected cells compared to 31–8000 VP/cell (r^2^, 0.9959 vs 0.9613) (Figure 1B). Therefore, the upper saturation limits of Ad5-Fluc and Ad5-EGFP were 8000 VP/cell and 1000 VP/cell, respectively. Each data point in Fig. 1A and B represented the average of three independent experiments, and the standard deviations for all points were lower than 0.98%.

The neutralizing activity of an Ad5-vaccinated mouse serum was compared using different virus concentration, 63,250, and 1000 VP/cell (Figure 1C–H). Approximately 40–60% virus neutralization was observed at VP/cell ratios of 63,250 and 1000 with naïve mouse serum during the serum dilution <30, but declined to <20% when the serum dilution was more than 30-fold (data not shown). A 250 VP/cell ratio (middle of the linear range) was chosen as the optimal virus infectious dose to perform the assay for a lower coefficient of variation compared to 63 VP/cell (p<0.05) (Figure 1D, E, G, H) and no significant difference between the Ad5NAb titers of two assays (1588±335 vs 1381±64, p = 0.32) (Figure 1I and J). Moreover, comparison of the addition of 5,000 cells/well and 30,000 cells/well in a 96-well plate showed that the higher cell density had better reproducibility at 250VP/cell, (CV, 7.1 vs 3.2% for CLNT and 5.2 vs 2.4% for FRNT) (Figure 1D and G). The final formats of the Ad5NAb assays for testing human sera are described in Table 1.

**Table 1:** Description of the final format of the CLNT and FRNT assays.

Step	Description
Samples collection	Collection by venipuncture. Inactivation of sera at 56°C for 30 min.
Dilution of sera	Preparation of seven 3-fold dilution steps in a 96-well plate in volume of 100 µl. Preparation of 150 µl medium for negative control. A control serum with established Ad5NAb titer was included as a standard.
Addition of virus	Dispensing of 50 µl containing 7.5×10^6^ VP of Ad5-Fluc or Ad5-EGFP to each well. Incubation at 37°C, 5%CO_2_ for 1 h.
Addition of cells	Addition of 100 µl containing 30,000 Vero cells to each well. Incubation at 37°C, 5%CO_2_ for 24 h.
CLNT measurement	Aspiration of 100 µl supernatant. Addition of 100 µl of Bright-Glo™ luciferin. Incubation at room temperature for 2 min, keeping from light. Measuring luminescence with GLOMAX™ 96 microplate luminometer.
FRNT measurement	The cells were trypsinized, resuspended in DPBS containing 1% FCS, and analyzed by FACS.

### Measurement of human Ad5NAb using CLNT and FRNT

At the positivity threshold of ≥30, the overall percentage of people with detectable Ad5NAb performed by CLNT was higher than for FRNT (85.4 vs 69.9%, p<0.001). Median Ad5NAb titers were >2-fold higher for the CLNT compared with the FRNT, and the average Ad5NAb (log_10_) titer in the 206 human samples measured by CLNT and FRNT assays were 2.83±0.84 and 2.45±0.84, respectively (p<0.001) (Table 2). As shown in Table 2, the percentage of serum samples positive for Ad5NAb significantly increased with age, from 54.2% to 77.8% with FRNT (p = 0.049) and from 70.8% to 92.6% with CLNT (p = 0.010). However, there was no significant difference in titer or prevalence by gender, 67.7% vs 72.0% (p = 0.503) and 84.8% vs 86.0% (p = 0.818), respectively. It was determined that the sera collected from Anhui Blood Center (BC) had a higher proportion of patients with Ad5NAb than Beijing BC, 78.3% vs 61.0% with FRNT (p = 0.007) and 91.5% vs 79.0% with CLNT (p = 0.011).

**Table 2:** Ad5NAbs measured by FRNT and CLNT assays.

Variables		Num.	Num. of cases (%)		Sig^a^	Mean of Ad5NAb (log_10_)^b^		Sig^c^
			FRNT	CLNT		FRNT	CLNT	
Age	18–20 years	48	26 (54.2)	34 (70.8)	0.008	2.28±0.85	2.61±0.94	<0.001
	21–30 years	49	35 (71.4)	43 (87.8)	0.008	2.47±0.80	2.84±0.79	<0.001
	31–40 years	55	41 (74.5)	49 (89.1)	0.008	2.57±0.90	2.96±0.85	<0.001
	41–56 years	54	42 (77.8)	50 (92.6)	0.008	2.48±0.78	2.88±0.75	<0.001
Sex	Male	99	67 (67.7)	84 (84.8)	<0.001	2.42±0.84	2.78±0.83	<0.001
	Female	107	77 (72.0)	92 (86.0)	<0.001	2.49±0.83	2.87±0.85	<0.001
Site	Beijing BC	100	61 (61.0)	79 (79.0)	<0.001	2.32±0.77	2.72±0.86	<0.001
	Anhui BC	106	83 (78.3)	97 (91.5)	<0.001	2.59±0.87	2.93±0.81	<0.001
Total		206	144 (69.9)	176 (85.4)	<0.001	2.45±0.84	2.83±0.84	<0.001

FRNT, Fluorescence-based NAb detection test; CLNT, Chemiluminescence-based NAb detection test.

a Significance calculated using Chi-square test (*p*-values).

b Values are expressed as mean±SD.

c Significance calculated using paired *t*-test (*p*-values).

### Comparison of CLNT and FRNT

**Figure 2:**
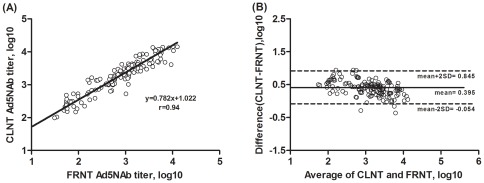
Comparison between CLNT and FRNT measurements. (A) Correlation between 50% Ad5 neutralizing antibody titers (log_10_) of 144 human sera positive by CLNT and FRNT assays. The line represents the fitted regression line. (B) Bland-Altman plot of Ad5NAb measurements using the CLNT and FRNT assays. Solid line indicates the mean value, and dashed lines represent 95% confidence limits. The x-axis indicates the average of Ad5NAb titers (log_10_) of CLNT and FRNT assays. The y-axis indicates the difference between the Ad5NAb titers (log_10_) of CLNT and FRNT assays.

Overall, there was 84.5% concordance between the two assays for the 206 samples tested; 144 positive by both assays and 30 negative by both assays. The FRNT could not detect Ad5NAb titers for 32 samples that were determined to be positive by the CLNT assay, with a range of titers of 30–300. For the 144 concordant positive samples, CLNT showed excellent correlation with FRNT (r = 0.94, p<0.001) (Fig. 2A). Using regression analysis, it was determined that Ad5NAb_CLNT_ = 0.782*(Ad5NAb _FRNT_)+1.022. Bland-Altman analysis revealed a close agreement (95.8%, 138/144) between the two assays: the results of the CLNT were on average 0.395 log_10_ higher than determined by the FRNT assay (Fig. 2B). Overall, the range for the mean difference between the quantitative values measured by the two assays (±2 standard deviations) was −0.054 to 0.845 log_10_. Quantitative differences outside the limits of agreement were only observed between CLNT and FRNT assays for six samples.

### Confirmation of NAb test with Western blot analyses

**Figure 3:**
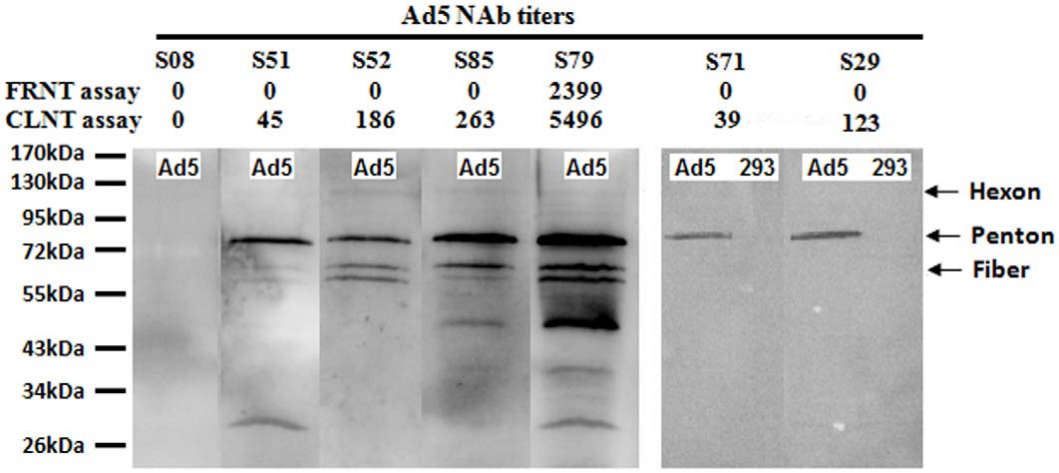
Confirmation of Ad5NAb by western blotting assay. Sample S08 was negative and S79 were positive for Ad5NAb titers for both assays. Samples S29, S51, S52, S71 and S85 were CLNT-positive/FRNT-negative sera. The uninfected HEK293 cells as negative control was used while two samples (S71, S29) were performed confirmation by western blotting assay. The Ad5 viral capsid is composed of three major types of proteins: hexon (130 kDa), penton base (82 kDa), and fiber (62 kDa).

For confirmation of the CLNT and FRNT results by western blot, 9 out of 32 CLNT-positive/FRNT-negative sera were randomly selected for analysis. The results obtained by neutralizing antibody and western blot methods were consistent. As shown in Fig. 3, the serum sample S79 with the highest Ad5NAb titer had the strongest antigen recognition by western blot, whereas the five CLNT-positive/FRNT-negative sera were confirmed positive by western blot but with weak Ad5 specific antigen recognition, with comparison to the uninfected HEK293 cells [20], [21]. Seronegative samples determined by both assays remained negative by western blot.

## Discussion

Recombinant Ad5-based gene transfer vector platforms are heavily utilized for both gene therapy and vaccine applications due to their well understood biology that includes excellent safety, high vector yields, and high transgene expression in a wide range of eukaryotic cells [22]–[24]. Their usefulness for permanent gene replacement is limited by their high immunogenicity, which results in the rapid elimination of transduced cells through induction of T and B cells to adenovirus antigens and the transgene product [11], [25]. Therefore, a qualified neutralization assay is required to investigate the baseline Ad5NAb serostatus for guiding the administration of Ad5 vector-based products.

To our knowledge, this is the first head-to-head comparison of the two high-throughput platforms, CLNT and FRNT, currently used in many studies. In this report we compared Ad5 neutralization assays to define an optimal assay based on the criteria of simplicity, speed, and sensitivity. The principle of two assays is the same: serum, transgenic reporter virus, and cells are incubated, which allows antibodies present in the serum to neutralize the virus, thereby inhibiting infection. Subsequently, inhibition of virus infection can be detected by transgene expression. To make the different assays easy to be compared, we had composed one protocol for the two assays that, in our view, represented the replication inhibition method commonly used. Critical parameters, virus inoculation dose, and cell density, were standardized to achieve reliable comparison of data from the two methods using Fluc and EGFP transgene reporters, respectively. The determination of optimal viral concentration is important because the infectious dose must be lower in order to increase assay sensitivity. However, the infectious dose must be high enough to produce a signal that will allow measurement of reduction due to neutralization.

In this assay, we determined that 250 VP/cell, which was determined to be in the middle of the linear range of detection, was appropriate for reproducibility and sensitivity. Similarly, Sprangers et al [14] selected a dose of 500 VP/cell for Ad5NAb experiments. The linear correlation obtained between the Ad5 vector concentration and Fluc transgene expression or percent of infected cells supports the hypothesis of one virion-one transduction event [26]. It was found that both human and mouse sera had nonspecific neutralization effects at 1∶30 dilution. We believe that this inhibition was mainly due to the decrease of Vero cell activity affected by various species of serum. In this assay, the samples were considered positive only when the inhibition percentage reached 50%. In fact, all naïve samples at the starting dilution (1∶30) were determined to be negative although their inhibition percentage ranged from 5–15%. Thus, an inhibition percentage <50% would not affect the results of detection. It was found that the nonspecific inhibition effect decreased as serum dilution increased from 30 folds. Furthermore, with comparison to the inhibition effect of specific Ad5NAbs, the nonspecific neutralization effect could be ignored when the serum dilution was greater than 30-fold.

The current study showed that the intermediate prevalence (69.9 and 85.4%, respectively for the two assays) determined in human sera obtained from the northern Chinese region was in agreement with that reported for the southern Chinese region using the CLNT assay and an Ad5 expressing a secreted alkaline phosphatase (77.3%) [15]. Moreover, these data were also consistent with those found for Ad5NAb seroprevalence in regions of the world with lower seroprevalence, such as Europe and the USA (35–70%) [9], [18], and regions with a higher prevalence, such as Africa and other Asian regions (75–100%) using CLNT or FRNT assay [12], [19]. As more Ad5 vector-based vaccines are evaluated in diverse populations around the world, geographic variation may be important in the design of future vaccine trials.

Interestingly, we found that the percentage of positive Ad5NAb samples and serum titers significantly increased with age, with 18- to 20-year-old individuals having the lowest levels of Ad5 immunity, which was consistent with the finding mentioned by Caijun Sun, indicating that the older population (41–72 years) in southern China had the highest seropositivity (84.8%) and percentage (54.4%) of the high Ad5 neutralizing antibody titers (>1000) [15]. This finding is likely to broaden the use of recombinant Ad5-based vaccines to this age group of <20 years. We believed that the increase was due to the change in the social behavior of individuals at this age, as when they move outdoors and have increased contact with others, thus having a higher chance of exposure to adenovirus.

The current study also determined that there was significant variation in the prevalence of Ad5NAb between the populations of donors from different geographic regions in China. This variation could be a consequence of different level of health care of individuals between the two locations studied. The prevalence of pre-existing Ad5NAb could guide the future administration of Ad5 vector-based products in Beijing and Anhui provinces. However, our current study has several limitations. Although a fairly large number of samples were obtained from the different sites, participant sampling was not random and, therefore, may not be representative of the general population. Taking the high level of pre-existing Ad5NAb into account, Ad5 vector-based products should be administered at higher dosage or via the intranasal route in future studies. Croyle et al [27] showed that nasal immunization with an Ad5-based vaccine can induce a long-term protective immune response against Ebola virus in a mouse model which was not impeded by pre-existing immunity to Ad5. Alternatively, Osada et al [28] explored a novel strategy that specifically reduced the expression of structural Ad5 genes by creating E1- and E2b-deleted recombinant Ad5 vectors to address the pre-existing Ad5NAb issue.

Comparison of the two current assays used for assessing Ad5Nab showed that the CLNT was significantly more sensitive than FRNT, although an excellent correlation and close agreement for the high titer of positive samples between the assays was found. Consistent with this finding, Yang et al [29] declared that the sensitivity of the chemiluminescence detection was 10-fold greater than a fluorescence-based assay, and 80- to 100-fold greater than a conventional colorimetric method. Maybe, one reason for the higher sensitivity in CLNT compared to FRNT was because the trypsinization of Vero cells might lead to increased lysis of infected cells, especially if they were carrying higher viral loads. To confirm whether the titers calculated using two assays were correlated with the actual pre-existing antibodies specifically recognizing Ad5 proteins, samples with different determined titers of Ad5NAb were analyzed by western blot. There was 100% agreement between neutralizing antibody and western blot assays. Interestingly, difference in the amount of antibody reactive to three major components of the viral capsid in human sera, (penton>fiber>hexon) may be determined by the order of appearance of antibodies during infection. Gahery-Segard et al [30] previously observed the sequential appearance of these antibodies with particle-coated enzyme-linked immunosorbent assay: anti-fiber antibodies appeared first, followed by anti-penton antibodies, and then by anti-hexon antibodies. However, Bradley et al [20] observed that neutralizing antibody responses to hexon appeared be more predominant than anti-fiber antibodies in sera from vaccinated mice, vaccinated humans, and naturally exposed humans. Cheng et al [21] demonstrated that rAd5 neutralizing antibodies were directed to different components of the virion, depending on whether they were elicited by natural infection or vaccination. The vaccine targeted more of the capsid antibody titer, whereby the natural infection had more specificity to the fiber. Notably, preexisting immunity to fiber significantly reduced the CD4 and CD8 cell responses to HIV Gag after rAd5 vaccination. In this study, we found the ratios of penton:fiber between S52 and S85, which had similar levels of Ad5NAb titers, were variable . So, it was strongly suggested that both the quantity and the specificity of Ad5 neutralizing antibodies should be focused on. Several recombinant Ad5 vectors have been engineered to circumvent pre-existing anti-Ad5 immunity by removing key neutralizing epitopes on the surface of viral capsid proteins, such as Ad5/35 fiber chimeras and Ad5/48 hexon chimeras [7], [23], [31]. Such chimeric viral vectors may have important practical implications for vaccination and gene therapy.

In summary, two simple, objective, reproducible, time- and labor-saving chemiluminescence-based and fluorescence-based neutralization assays were developed for the determination of Ad5NAb titers in human sera. Significantly different levels of Ad5NAbs were measured in samples by the two assays. Therefore, due to its sensitivity and simplicity, it is suggested that the CLNT is the preferred method to be used for future epidemiological studies of Ad5NAb in other localities.

## Methods

### Ethics issues

The study design was inspected and approved by Ethical Committee of Beijing Blood Center. Each participant was informed of the purpose of the study and written consent was obtained from each participant involved in this study.

### Cells

Vero (ATCC, CCL81) and HEK293 (ATCC, CRL-1573) cells were used for the CLNT and FRNT neutralizing assay, grown at 37°C under 5% CO_2_ in Dulbecco's modified Eagle's medium (DMEM) supplemented with 10% fetal bovine serum (FBS), 1% L-glutamine, 1% combined antibiotics and 1% non-essential amino acids (HyClone, South Logan, UT, USA).

### Sera samples

Serum samples from 206 donors (99 males, 107 females) ranging in age from 18–56 years as of 2011 were obtained from a diverse human population of healthy individuals in Beijing and Anhui province, China, with no known prior exposure to Ad5 virus. The samples from healthy participants were collected from the Beijing BC (100 samples) and Anhui BC (106 samples). There was roughly a 1∶1 male:female in each age group in both Beijing and Anhui participants. All samples were frozen at −80°C and banked at the institutions after collection. Samples were shipped on dry ice to the National Institutes for Food and Drug Control. All samples were thawed, aliquoted, and stored at −80°C until assayed. All samples in our study were subjected to identical treatment and storage conditions, and were considered of equivalent quality.

### Ad5-Fluc and Ad5-EGFP viruses

Using the Fluc and EGFP genes derived from pLUCF and pDRVI1.0-EGFP, respectively (kindly provided by John T. Schiller, National Cancer Institute, Bethesda, MD, USA and Jianqing Xu, Chinese Center for Disease Control and Prevention, Beijing, China), the Ad5-Fluc and Ad5-EGFP were constructed according to the AdMax method [32]. Briefly, PCR was used to construct recombinant shuttle plasmid pDC316-Fluc and pDC316-EGFP. HEK293 cells were cotransfected with structural gene expression plasmids (pBHGlox-E1,3Cre) and the reporter plasmids for Ad5-Fluc and Ad5-EGFP construction using Lipofectamine 2000 (Invitrogen, Carlsbad, CA). Recombinant vectors were serially-passaged on HEK293 cells to generate high-titer viral stocks.

### CLNT and FRNT based Ad5 neutralization assays

Ad5NAb was quantified using the chemiluminescence- assay and fluorescence-based assays according to established methods in which the neutralization was measured by the reduction in Fluc reporter gene expression of recombinant Ad5-Fluc virus and in percentage of EGFP protein expression positive cells inhibited by Ad5NAb present in serum samples, respectively. During the development stages of the assay, the following parameters were tested: cell density and inoculum dose of virus. Cells density was tested with 5,000 or 30,000 cells/well in a 96-well flat-bottom culture plate (Corning-Costar, Tokyo, Japan). In determining the optimal viral dose for the assay, we considered both the natural infectivity of the virus in Vero cells and the variability of infection in the presence of varying dilutions of serum containing Ad5NAb, involving 63,250, and 1000 VP/cell of diluted viruses and nine dilutions of anti-Ad5 mouse serum (AAMS), which was collected at 2 weeks after the second Ad5 empty virus inoculation. The diluted recombinant Ad5 viruses (50 µL) and serial three-fold dilutions of sera to be tested starting at 1∶30 (100 µL) were mixed in each of three corresponding wells in a 96-well plate, then incubated at 37°C, 5% CO_2_ for 1 h. Vero cells were added as a trypsinized single-cell suspension. The assay was incubated for 24 h after infection. To monitor inter-assay variability, there were at least two control wells that contained cells plus 150 µL of medium, acted as blank samples for detection and deduction of the background.

For the CLNT assay, 100 µL of supernatant was aspirated and 100 µL of D-luciferin substrate (Caliper, Hopkinton, MA, USA) was added into each well protected from light at room temperature for 2 min, after which luminescence was measured using a GLOMAX 96 microplate luminometer (Promega, Madison, WI). the 50% neutralization titer (NT_50_) for each serum sample was defined as the serum dilution at which the relative light unit (RLU) was reduced by 50% compared with virus-containing control wells after subtraction of the background RLU in cell-containing control wells.

For the FRNT assay, the cells were trypsinized, resuspended in DPBS containing 1% FCS, and analyzed by FACS (BD, Franklin Lakes, NJ) to evaluate the percentages of positive cells. The NT_50_ of each serum was defined as the reciprocal of the highest dilution that reduced the rate of transduced cells by 50% in comparison with wells containing virus but without antibody. Titers of ≥30 were accepted positive.

### Confirmation of NAb result by western blot analysis

As previously reported, the purified Ad5 viruses (10^10^ VP) were lysed with 1% (v/v) Triton X-100, resuspended in Laemmli lysis buffer, boiled for 5 min, and subjected to SDS-PAGE and then transferred to polyvinylidene fluoride membrane (Millipore, Billerica, MA) [15]. After blocking, the membrane was incubated for 1 h at room temperature with an equal volume of serum, at a 1∶500 dilution, from participants with either negative, low or high Ad5NAb titers, as determined by CRNT or FRNT. Then, the membrane was incubated with horseradish peroxidase-conjugated goat anti-human IgG antibody (Promega, Madison, WI) at a 1∶2500 dilution for 1 h. Finally, antibody reactivity was revealed using the ECL system (GE Healthcare, Piscataway, NJ).

### Statistical analysis

The Ad5NAb titer levels in the positive samples measured by two assays were compared using correlation and Bland-Altman analyses. Comparisons between the Ad5NAb seroprevalence in different assays and in different groups were evaluated using the Chi-square test, and differences in means were assessed using the Student's *t*-test available in SPSS (ver. 18.0; IBM, Armonk, NY). A p value of <0.05 was considered significant.
